# Nature-inspired chiral metasurfaces for circular polarization detection and full-Stokes polarimetric measurements

**DOI:** 10.1038/s41377-019-0184-4

**Published:** 2019-08-28

**Authors:** Ali Basiri, Xiahui Chen, Jing Bai, Pouya Amrollahi, Joe Carpenter, Zachary Holman, Chao Wang, Yu Yao

**Affiliations:** 1 School of Electrical, Computer and Energy Engineering, Arizona State University, Tempe, AZ 85281 USA; 2 Centre for Photonic Innovation, Arizona State University, Tempe, AZ 85281 USA; 3 Biodesign Centre for Molecular Design & Biomimetics, Arizona State University, Tempe, AZ 85281 USA

**Keywords:** Optics and photonics, Electronics, photonics and device physics

## Abstract

The manipulation and characterization of light polarization states are essential for many applications in quantum communication and computing, spectroscopy, bioinspired navigation, and imaging. Chiral metamaterials and metasurfaces facilitate ultracompact devices for circularly polarized light generation, manipulation, and detection. Herein, we report bioinspired chiral metasurfaces with both strong chiral optical effects and low insertion loss. We experimentally demonstrated submicron-thick circularly polarized light filters with peak extinction ratios up to 35 and maximum transmission efficiencies close to 80% at near-infrared wavelengths (the best operational wavelengths can be engineered in the range of 1.3–1.6 µm). We also monolithically integrated the microscale circular polarization filters with linear polarization filters to perform full-Stokes polarimetric measurements of light with arbitrary polarization states. With the advantages of easy on-chip integration, ultracompact footprints, scalability, and broad wavelength coverage, our designs hold great promise for facilitating chip-integrated polarimeters and polarimetric imaging systems for quantum-based optical computing and information processing, circular dichroism spectroscopy, biomedical diagnosis, and remote sensing applications.

## Metasurfaces: Inspired by nature

Inspired by the polarization-sensitive vision of the compound eyes in a marine crustacean called the Mantis Shrimp, researchers from Arizona State University, US have designed a chiral metasurface for manipulating the polarization of light. The metasurface design consists of a thin nanostructured silicon layer, a dielectric spacer layer and a gold nanowire polarizer, and has a total thickness of less than 1 micrometer. This thin planar surface offers low optical loss with a transmission as high as 80% in the near-infrared wavelength range, and acts as a circular polarization filter with an extinction ratio as high as 35. The circular polarization filters, in combination with linear polarization filters, can enable chip-scale polarimeters for sensing the polarization state of light. This on-chip integrated approach could prove useful in ultra-compact devices for advanced imaging and sensing applications.

## Introduction

Circularly polarized light (CPL) has been widely used in quantum communication^1^, quantum computing^2,3^, circular dichroism (CD) spectroscopy^4^, and polarimetric imaging and sensing^5–7^. Traditionally, CP light detection requires multiple bulky optical elements such as polarizers, waveplates, and mechanically rotating components^8^, which poses fundamental limitations for device miniaturization, robust system integration, and high-speed operation. Organic chiral molecules have been proposed for miniaturization of CPL detection devices, such as liquid crystals (LCs)^9,10^, chiral dyes^11^, and helicene-based chiral semiconductor transistors^12^. Recent developments in nanotechnology and nanophotonics have enabled ultracompact solid-state CPL detection^13–23^ (see Supplementary Table S1). Compared with organic chiral molecules, nanostructure-based devices generally exhibit superior stability in ambient conditions, fast response time, and high fidelity. For example, artificial three-dimensional (3D) metamaterials have been produced based on chiral L-shaped^22^, helical^20,24–26^, and spiral^21^ nanostructures to differentiate the handedness of CPL. However, the fabrication of these complex 3D structures requires stringent process control, and scalability is challenging. More recently, planar (or 2D) chiral plasmonic metasurface structures composed of gammadions^16^, Z-shaped antennas^17^, spiral slots^18^, and even stacks of twisted planar and achiral structures (crosses^14^, nanorods^15,23^, etc.) with chiro-optical responses have been reported. Compared with 3D chiral metamaterials, metasurface structures are easier to fabricate and more compatible with on-chip manufacturing technologies; however, the chiral plasmonic metasurfaces experimentally demonstrated so far usually suffer from low circular polarization extinction ratios (CPERs) (less than ~ 5) and limited optical efficiency in experiments (20–50%). To improve the optical efficiency, chiral dielectric metasurface structures have been achieved in experiments^13,19^ with optical efficiencies as high as 90%. However, the CPERs of these surfaces are still limited (up to eight). To date, the realization of ultracompact CPL detection devices with simultaneously high extinction ratios and optical efficiencies is still challenging.

In addition to CPL detection, ultracompact polarimetric detection and imaging systems are highly desirable for full polarization state measurements in various applications such as communication^27,28^, remote sensing^29^, polarization imaging^30^, and biological diagnostics^31–33^. Among different polarimeter designs (Table 1), plasmonics-based polarimetric detecting devices have been reported with unprecedented compactness based on phase-gradient birefringent metagratings^34^, diffracting plasmonic metasurfaces^35^, and graphene-integrated anisotropic metasurfaces^36^. However, these devices operate in reflection mode and hence are not compatible with direct on-chip integration in photodetectors or imaging sensors. Polarization-dependent surface plasmon polariton (SPP) structures have been demonstrated with feasibility for direct integration in detectors or imaging sensors^37–39^; however, these devices suffer from low efficiency. Very recently, a highly efficient phase-gradient all-dielectric metasurface polarimeter^40^ was reported with a high efficiency (60–65%). This approach enables splitting and focusing of light in three different polarization bases in transmission mode and provides a feasible method to realize on-chip polarimetric imaging arrays. However, the polarization measurement accuracy is fundamentally limited by the crosstalk between different polarization states and noticeably degrades as the super-pixel size becomes smaller than 7.2 µm.

**Table 1:** Full-Stokes polarimetric imaging techniques

Structure design	Operation mode	Operational wavelength	Efficiency	Error of Stokes parameters (average)
Plasmonic metagrating^34^	Reflection, diffraction	700–1000 nm	<50%	~10%
Diffracting integrated plasmonic metasurfaces^35^	Reflection, diffraction	500–700 nm	36–55% (numerical)	NA
Graphene-integrated anisotropic metasurfaces^36^	Reflection	6.7–6.8 μm	~10%	>3.9% (S1), >6.5% (S2), >2.5% (S3)
SPP excitation by X-shaped aperture array^38^	Transmission	750–1050 nm	<4%	7.3–12.3% (S1), 7.2–27.4% (S2), 5.2–17.7% (S3)
Integrated plasmonic polarimeter^39^	Absorption	~ 830 nm	NA	~45%
Metasurface in-line polarimeter^37^	Scattering	1500–1565 nm	NA	6% (S1), 5.8% (S2), 4.7% (S3)
Spin–orbit interaction of light with scatterers^50^	Transmission	1.5–1.6 μm	NA	7.4% (S1), 15.6% (S2), 11.4% (S3)
Dielectric metasurface^40^	Transmission, diffraction	845–855 nm	60–65%	7.5–15%
This proposal: metal-dielectric hybrid (ODLM)	Transmission	1.4–1.55 μm	~80%	1.9% (S1), 2.7% (S2), 7.2% (S3)

Reported errors for the Stokes parameters are the arithmetic mean (Ra) extracted from the data presented in each article

Inspired by the compound eyes of stomatopods^41,42^, in this paper, we theoretically and experimentally demonstrated double-layer chiral metasurface structures for near-infrared wavelength polarimetric detection with CPERs as high as 35 and transmission efficiencies >80%. The structure consists of a low-loss dielectric metasurface, an oxide spacer layer, and a nanowire polarizer, with a total thickness of less than 1 µm. In addition to CPL detection units, we integrated the chiral metasurface structures on the same chip with linear polarization (LP) filters to perform full-Stokes polarimetric detection. Our designs are advantageous due to the feasibility of direct and scalable integration onto existing imaging sensors, high extinction ratios, high transmission efficiencies, ultracompact footprint (subwavelength thickness, micrometer scale in the lateral dimension), and robustness; thus, these designs are ideal for ultracompact imaging, sensing, communication, and navigation systems.

## Results

### Design

**Fig. 1:**
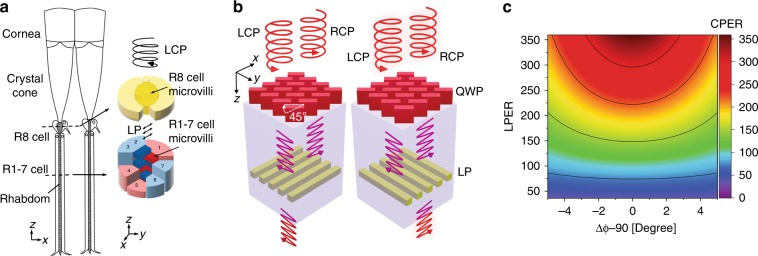
Bioinspired chiral metasurface design. **a** Anatomical schematic of ommatidium in the compound eye of mantis shrimp responsible for circularly polarized light detection. Left side shows the longitudinal cross section of the midband in the compound eye. Right side shows the transverse cross section of the R8 and R1–7 cells along the dashed line in the longitudinal cross section. **b** Schematics of ommatidium-like double-layer metamaterial (ODLM) design, where the dielectric metasurface behaves as artificial R8 cells and the nanogratings behave as R1–7 microvilli that differentiate the linear polarization perpendicular or parallel to the microvilli axis. **c** Theoretically calculated CPER based on Jones calculus, as a function of the relative phase difference between the fast and slow axes of the metasurface QWP and LPER of the polarizing nanograting. **a** was adapted with permission from Nature Publishing Group^42^

In nature, stomatopods (or mantis shrimps) possess extraordinary circular polarization vision due to the unique ommatidium designs in the midband of their eyes^42,43^. Each ommatidium, as shown in Fig. 1a, has a top retinular cell (R8) and seven bottom retinular cells (R1–7). The R8 cell acts as a quarter waveplate (QWP) to convert CPL to linearly polarized light (LPL). The microvilli of the R1–7 cells function as wire-grid polarizers that are oriented 45° with respect to the long axis of the R8 cell to discriminate converted LPL of different orientations. Inspired by this unique natural design, we created vertically integrated double-layer metasurface designs that mimic the ommatidium of stomatopods to distinguish CPL with different handedness. The ommatidium-like double-layer metasurface (ODLM) design is composed of a nanostructured birefringent metasurface acting as a QWP^44^, a linear polarizing nanograting, and a dielectric spacer layer between these components, as shown in Fig. 1b. The birefringence of the metasurface is achieved with an optically isotropic material, e.g., silicon, by patterning this material into nanostructures with structural anisotropy. Importantly, although neither the top metasurface nor the bottom linear polarizer is chiral, the combination of the two materials results in a hybrid chiral metasurface structure with no inversion center or reflection symmetry. Here, we introduce an angle of ±45° between the fast axis of the metasurface QWP and the axis of the lower polarizing nanograting; thus, the mirror symmetry is broken in these double-layer chiral metasurface structures.

To obtain a quantitative description of the device working principles without tedious full-wave simulations, we set up a simplified model of the proposed ODLM structure based on Jones matrices and obtained the transmissions corresponding to left-handed (LCP Jones vector $$\frac{{\sqrt 2 }}{2}\left[ {\begin{array}{*{20}{c}} 1 \\ i \end{array}} \right]$$) and right-handed incident CP light (RCP $$\frac{{\sqrt 2 }}{2}\left[ {\begin{array}{*{20}{c}} 1 \\ { - i} \end{array}} \right]$$) (for more details, see the Supplementary Information section 1):

1 $$\begin{array}{l}T_{{\mathrm{LCP}}} =\frac{1}{4}\left( {\left| {\gamma _x^{V/H}\gamma _fe^{ - i(\frac{{\Delta \varphi }}{2} + \frac{\pi }{4})} + \gamma _x^{V/H}\gamma _se^{i(\frac{{\Delta \varphi }}{2} + \frac{\pi }{4})}} \right|^2 + \left| { - \gamma _y^{V/H}\gamma _fe^{ - i(\frac{{\Delta \varphi }}{2} + \frac{\pi }{4})} + \gamma _y^{V/H}\gamma _se^{i(\frac{{\Delta \varphi }}{2} + \frac{\pi }{4})}} \right|^2} \right)\\ T_{{\mathrm{RCP}}} =\frac{1}{4}\left( {\left| {\gamma _x^{V/H}\gamma _fe^{i( - \frac{{\Delta \varphi }}{2} + \frac{\pi }{4})} + \gamma _x^{V/H}\gamma _se^{ - i( - \frac{{\Delta \varphi }}{2} + \frac{\pi }{4})}} \right|^2 + \left| { - \gamma _y^{V/H}\gamma _fe^{i( - \frac{{\Delta \varphi }}{2} + \frac{\pi }{4})} + \gamma _y^{V/H}\gamma _se^{ - i( - \frac{{\Delta \varphi }}{2} + \frac{\pi }{4})}} \right|^2} \right)\end{array}$$

where Δ*φ* is the phase retardation introduced by the QWP (note the fast axis is oriented −45° with respect to the *x*-axis) and *γ*_*f*_ and *γ*_*s*_ are the transmission coefficients of the QWP for the electric field components along the fast and slow axes, respectively. $\gamma _x^{V/H}$ and $\gamma _y^{V/H}$ are the transmission coefficients of the polarizing nanograting for the electric field components along the *x* and *y* axes, corresponding to the vertical (*V*) or horizontal (*H*) nanograting orientation, respectively. We apply equation (1) to calculate the dependence of the CPER on the performance of the QWP and linear polarizer, as shown in Fig. 1c. The results indicate that the maximum CPER occurs at $\Delta \varphi = \frac{\pi }{2}$ and is ultimately limited by the extinction ratio of the linear polarizer (${\mathrm{LPER}} = \gamma _i^{V/H}/\gamma _j^{V/H}$ for gratings oriented along the *j*-axis, *i*, *j* = *x*, *y*). Therefore, to achieve a high CPER, we need to maximize the LPER and design a perfect QWP with $\Delta \varphi = \frac{\pi }{2}$.

**Fig. 2:**
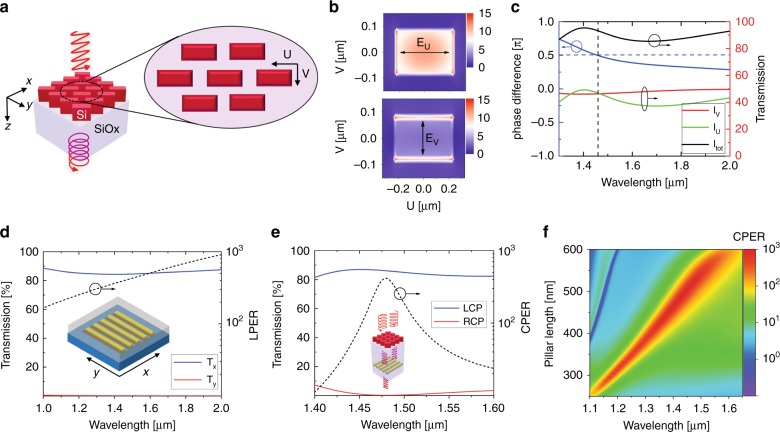
Design of ODLM-based CPL filters. **a** Schematics of silicon quarter waveplate. *U* and *V* are the axes corresponding to the long and short axes of the nanopillars. **b** Near-field distribution in the middle of the metasurface when the incident polarization is along the length (top) and width (bottom) of the unit cells. **c** Phase delay difference between the *U* and *V* axes (left axis), corresponding transmissions along the *V* (red) and *U* (green) axes and total transmission (black) (right axis). **d** Transmission through the gold nanograting immersed in silicon oxide (left axis) and the corresponding LPER (right axis). **e** Transmission (left) and CPER (right) of ODLM device. **f** Wavelength tuning of device by sweeping the length of the silicon nanopillars (and hence the aspect ratio) while all the other parameters are fixed. The length and width of the designed metasurface QWP are 480 and 160 nm, respectively. The thicknesses of the SiO_*x*_ spacer, Si, and SiO_*x*_ hard mask layers are 350, 700, and 100 nm, respectively. The nanograting width is 84 nm, and the thickness is 195 nm

We choose dielectric metasurfaces composed of silicon nanopillars with geometrically induced birefringence (Fig. 2a) to realize QWPs with accurate phase retardation control and high transmission efficiency. The Si nanopillars are designed with subwavelength dimensions (length 480 nm, width 160 nm, and height 700 nm) to avoid Mie resonance. The birefringence of the silicon nanopillar array results from the distinct near-field distributions for different incident light polarization^45–47^, as illustrated in Fig. 2b. For incident LP light polarized along the slow axis (*U*-axis) of the metasurface QWP, the electric field intensity is mostly localized in silicon (top panel in Fig. 2b). In contrast, for incident light polarized along the fast axis (*V*-axis) of the metasurface QWP, the electric field intensity is mostly located in the air gaps between the Si pillars (bottom panel in Fig. 2b). As a result, the metasurface QWP phase retardation between the fast and slow axes can be precisely engineered by adjusting the metasurface geometry to be exactly *π*/2 at the operation wavelength, e.g., at 1.47 µm, as shown in Fig. 2c. Moreover, the transmission coefficients are designed to be the same for the LP components along both the fast and slow axes, with a total transmission efficiency over 90% around the operation wavelength. The linear polarizer design is composed of gold nanowires with optimized dimensions (period 230 nm, duty cycle 37%, and thickness 195 nm) to achieve an LPER greater than 200 and transmission efficiency >85% for the wavelength range from 1 to 2 µm (Fig. 2d). More details about the dependence of the nanograting performance on the geometric parameters is included in the Supplementary Information (Fig. S2).

Although the Jones matrix model provides an intuitive physical model to illustrate the device physics and analytical results to assist in device design, this model is too simplified to consider multiple reflections inside the thin film structure and the near-field interactions between the metasurface QWP and polarizing nanograting. To provide an accurate performance analysis of the ODLM-based CPL filters proposed, we performed full-wave simulations to obtain the transmission spectra of the ODLM structure for LCP and RCP incident light. Figure 2e shows the results of an ODLM-based LCP filter designed at the operation wavelength of *λ*_0_ = 1.47 µm. The maximum CPER is greater than 400 with a transmission efficiency greater than 85% at the operation wavelength. This design can operate over a wavelength range of more than 100 nm (from 1.45 to 1.55 µm) with CPER over 30 and transmission efficiency over 80%. The operation wavelength *λ*_0_ of the ODLM CP filters can be tailored by changing the design parameters. We obtain devices with operation wavelengths from 1.1 to 1.65 μm by simply varying the length-to-width aspect ratio of the silicon pillars, as shown in Fig. 2f. The operation wavelength can also be engineered by adjusting the pillar height and the silicon volume filling factor (see Supplementary Information Fig. S1). Such engineering flexibility can enable on-chip wavelength multiplexing of the ODLM-based CPL filters with optimized device performance over broadband wavelengths.

### Device fabrication

**Fig. 3:**
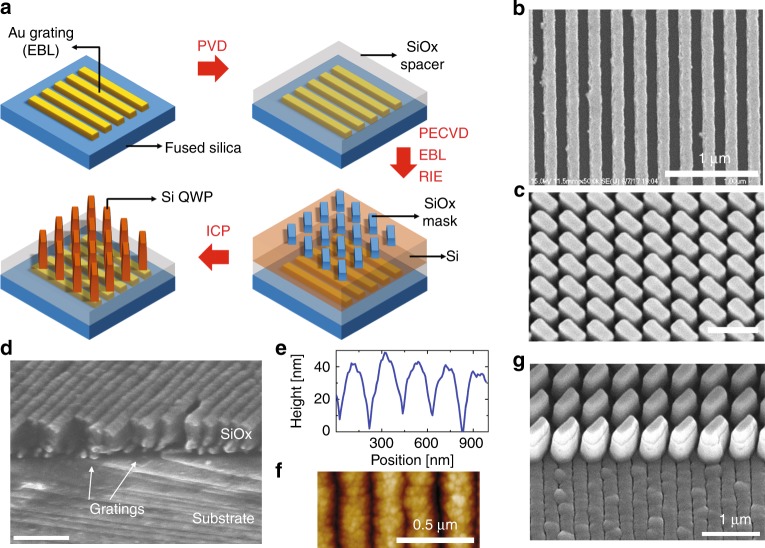
Fabrication of ODLM-based CPL filters. **a** Schematic illustration of major fabrication steps. **b** Scanning electron microscope (SEM) image of the nanograting before QWP metasurface integration. **c** SEM image of silicon QWP. **d** Cross section of grating covered by fused silica. The fringes on the surface of the SiO_*x*_ spacer layer reflect the underlying grating thickness and duty cycle. **e**, **f** Atomic force microscope (AFM) images of the nanograting after PVD deposition of SiO_*x*_ (~350 nm), indicating mitigated surface roughness (Ra = 11.5 nm). **g** Tilted-view SEM image of ODLM structure. The bright thin layer on top of each Si QWP pillar corresponds to residual SiO_*x*_ mask. All the SEM scale bars are 1 µm, and the AFM scale bar is 0.5 µm

The major challenge in the fabrication of ODLM-based CPL filters is to reliably integrate metasurface QWPs and the polarizing nanograting without degrading the optical performance or affecting the overall structural stability. Moreover, the fabrication process involves multiple steps of lithography, film deposition, and etching to form the gratings, the spacer layer, and the QWPs and therefore must be systematically designed to avoid structural integration issues, such as etching damage, poor film adhesion, high surface roughness, and feature distortion. Figure 3a illustrates the fabrication procedures we used to demonstrate the ODLM structures. First, gold nanogratings were fabricated on fused silica substrates by electron-beam lithography (EBL), metal evaporation, and metal liftoff. Then, a dielectric spacer layer (350 nm) of SiO_*x*_ was sputtered to cover the nanogratings, followed by plasma-enhanced chemical vapor deposition (PECVD) of *α*-silicon (*α*-Si). The *α*-Si layer was patterned into nanopillars by EBL and inductively coupled plasma reactive-ion etching (ICP RIE) using an SiO_*x*_ hard mask. The detailed fabrication steps are presented in the “Materials and methods” section and Supplementary Information (Fig. S3). Figure 3b shows a scanning microscope (SEM) image of the fabricated polarizing nanograting composed of 120-nm-thick gold nanogratings with a 230 nm period and 50% duty cycle. The nanogratings demonstrated were thinner than the optimized designs due to challenges in fabricating thicker nanogratings with low defect density. The use of metallic nanogratings allows a high LPER but also poses certain integration challenges because of instability of metal under elevated temperatures, poor adhesion to dielectrics, and rough surface. Here, we demonstrated that the use of a sputtered SiO_*x*_ dielectric spacer layer resolves or mitigates these problems. The nanograting samples were baked at a moderate temperature to remove the surface water molecules and in situ sputter-cleaned to remove the contaminants on the surface immediately before a room temperature sputtering process to cover the nanogratings with an SiO_*x*_ dielectric layer. The sputtered SiO_*x*_ spacer layer exhibited excellent adhesion to the fused silica substrate, filled the trenches in the nanogratings^48^, and greatly reduced the surface roughness resulting from the embedded nanogratings. As inspected by cross-sectional SEM (Fig. 3d) and atomic force microscopy (Fig. 3e, f), sputtering a 350 nm silicon oxide spacer layer onto the nanograting surface significantly decreased the surface roughness (Ra = 11.5 nm, as shown in Fig. 3e, f). The reduced surface roughness was crucial to the subsequent fabrication of Si nanopillar QWPs. Fourier transform infrared spectroscopy (FTIR) measurements and full-wave simulation results show that the SiO_*x*_-coated nanogratings experience only a slight degradation of the LPER (Supplementary Information Fig. S4), indicating that the dielectric layer fabrication process only minorly impacted the nanograting performance. In addition to the structural importance of the SiO_*x*_ spacer, the spacer helps to minimize the near-field interactions between the nanogratings and QWPs and ensure reliable device operation. Full-wave simulation shows that the optimal thickness is between 300 and 400 nm for our design in the 1.3–1.5 µm wavelength range (Supplementary Information Fig. S5).

Finally, the Si metasurface QWPs were aligned on top of the polarizing nanogratings. The metasurface QWP is composed of densely packed silicon nanopillars, as shown in Fig. 3c (length 480 nm, width 160 nm, and thickness 700 nm). The nanoscale dimensions and large height–width aspect ratios (HWAR > 4) pose significant challenges in fabrication. We used a double-layer hard mask (60 nm Cr on top of 160 nm of SiO_*x*_) to pattern the silicon nanopillars with the nanoscale geometries required. A 60-nm-thick Cr mask fabricated by EBL and metal liftoff was used to pattern the SiO_*x*_ mask. Then, the SiO_*x*_ layer served as a second hard mask for ICP RIE etching of *α*-Si nanopillars with a large HWAR. More details are provided in the “Materials and methods” section. Noticeably, our optimized fabrication process enabled etching of the nanopillars with steep sidewalls (<8° tilt angle, Supplementary Information Fig. S6), a lateral pillar gap as small as 200 nm, and an HWAR > 4 (Supplementary Information Fig. S3). To consider the sidewall inclination angle of the silicon nanopillar, we modified the structure dimensions in the numerical model and performed structure optimization accordingly to determine the device geometries (see Supplementary Information Fig. S7). Our theoretical analysis suggests that an inclination angle from 0° to 10° can achieve high CPERs (>300) at any desired operational wavelength.

### Device characterization and discussion

**Fig. 4:**
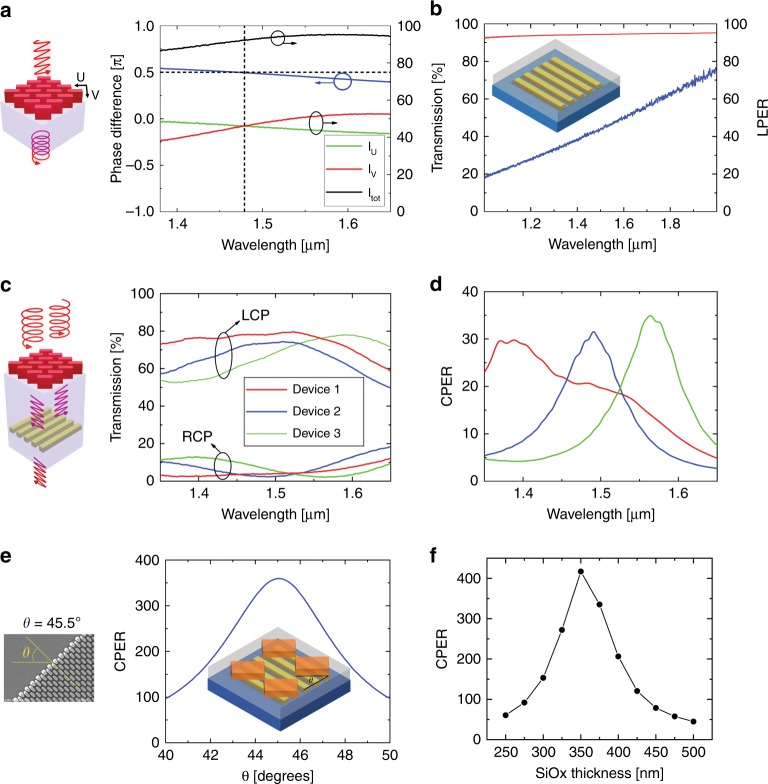
Characterization of ODLM-based CPL filters. **a** Phase delay (left axis) and transmission spectrum (right axis) of a fabricated QWP. **b** Transmission measured perpendicular to the nanograting orientation (red curve, left axis) and the corresponding linear polarization extinction ratio (blue curve, right axis). **c** Transmission spectra measured for three devices (designed to operate at three different wavelengths) for LCP and RCP incident light. **d** Extracted CPER for three devices presented in **c**. The dimensions measured (length, width, and period, in nanometers) for three devices are 403, 138, and 383 nm (red curve); 438, 153, and 418 nm (blue curve); and 480, 170, and 460 nm (green curve). **e** Analytically calculated CPER dependence of ODLM device on orientation angle between top QWP and bottom nanograting. The SEM measurement on the left panel shows that the error is less than 0.5°. **f** FDTD simulation of the CPER dependence on the fused silica spacer layer thickness, corresponding to an optimum value of ~350 nm

The performance of the metasurface QWPs and linear polarizing nanogratings were first characterized using FTIR (see “Materials and methods” section). To characterize the metasurface QWPs, LP light was directed incident to the device under testing with the electric field vector oriented at 45° with respect to the slow (*U*-) axis of the QWP. The state of polarization (SoP) of the transmitted light was measured with a rotating linear polarizer (polarization analyzer (PA)) to extract the phase retardation. The phase retardation and transmission spectra measured for the metasurface QWP designed within the telecom wavelength bands are shown in Fig. 4a. At the operation wavelength (1.47 µm), the phase retardation between the fast (*V*-) and slow (*U*-) axes is exactly π/2, and the transmission efficiency of the QWP is close to 90%. The nanograting linear polarizer fabricated (width 120, height 122, and period 230 nm as fabricated) exhibited a LPER of 40–50 and transmission efficiency > 90 at wavelengths of ~1.5 µm (Fig. 4b). We attribute the lower LPER mainly to the nanograting thickness (120 nm) being smaller than that of the optimized device designs (190 nm) (see Fig. S2a). The chip-integrated ODLM-based CPL polarization filters were characterized with both LCP and RCP input light, as illustrated in the schematic in Fig. 4c (see “Materials and methods” section). Figure 4c shows the transmission spectra measured for three devices fabricated on the same substrate for both LCP and RCP input light in proximity to the operation wavelengths of the devices. These devices were designed for different operation wavelengths from 1.4 to 1.6 µm. The devices all have high transmission efficiency (70–80%) for LCP light and minimal transmission (2–3%) for RCP light. The maximum CPERs (defined as *I*_LCP_/*I*_RCP_) for all three devices are over 30, as shown in Fig. 4d. All of the devices provide CPERs of more than 10 over a wavelength range of 150 nm at approximately the operation wavelength. Compared with other on-chip solid-state-based CPL filters reported in the literature, the ODLM-based CPL filters provide the best performance when taking both CPER and efficiency in consideration (see Supplementary Information Table S1).

Compared with the simulation results of the optimized device design, there is still much room for improvement of the CPERs of the ODLM-based CPL filters. Due to the limitations of the nanofabrication process, the devices experimentally demonstrated deviation from the simulations in a number of aspects. In addition to the imperfections of the fabricated nanogratings and silicon metasurfaces discussed previously, the alignment errors between the metasurfaces and the nanogratings underneath affect the device performance. Figure 4e shows the dependence of the CPER on the error in the rotational alignment between the slow axis of the metasurface QWP and the LP polarizer. From the SEM images of the samples fabricated, we estimated a degree error of less than 0.5 from the ideal value (45°) (Supplementary Information Fig. S8). According to the results shown in Fig. 4e, this angle deviation leads to a slight decrease (<6%) in the CPER of the CP filter. Further, the translational displacement between the silicon pillars in the QWP and the nanowires in the linear polarizer only slightly affected the performance of the ODLM structure design (<2%, Supplementary Information Fig. S9). In addition, we studied the dependence of the CPER on the dielectric spacer layer thickness at the working wavelength (Fig. 4f) and found a reasonably small degradation of the CPER (<5%) for a deviation of 5 nm from the spacer thickness designed (350 nm). Based on the experimental evidence and theoretical analysis, we conclude that the key practical limitations in the double-layer integration do not significantly affect the CPERs of the ODLM-based CPL filters fabricated. However, significant improvement of the device performance could be achieved by improving the LPER of the polarizing nanogratings. Our numerical simulations reveal that a change in the nanograting thickness from 120 to ~190 nm increases the LPER from ~50 to over 400.

**Fig. 5:**
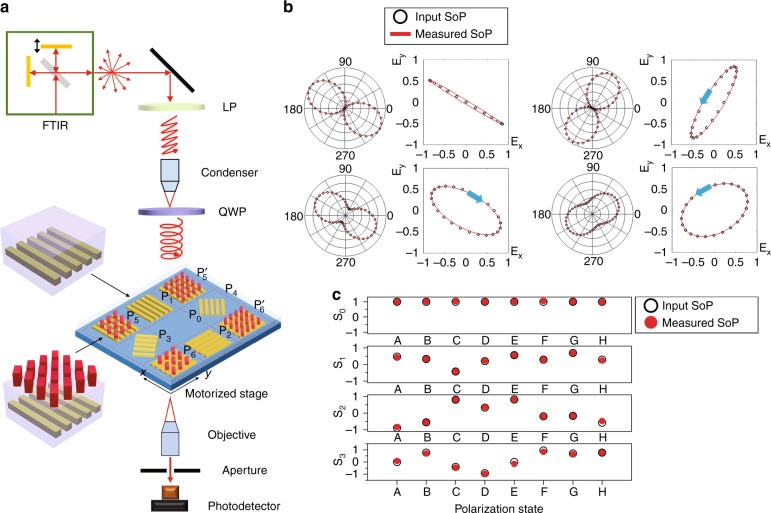
Full-Stokes polarimetric measurements. **a** Schematics and optical microscope image of metasurface design to fully characterize the polarization state (SoP) of the incident light. The required units are marked by P_0_ through P_6_. The two additional units labeled ${\mathrm{P}}_5^\prime$ and ${\mathrm{P}}_6^\prime$ provide the capability to identify Stokes parameters at a different working wavelength. **b** Comparison of the SoP obtained using a conventional polarization analysis method (black circles) and our device (red solid lines), using polar plots and polarization ellipses, at an operational wavelength of 1.45 µm. **c** Stokes parameters (S_0_–S_3_) extracted for eight different random input SoPs, using a polarization analyzer (black circles) and our device (red circles)

To demonstrate the concept of on-chip polarimetric detection, we integrated an array of polarization filters on the same chip, including four LP filters and two ODLM-based CP filters, for full-Stokes polarization state measurements based on a spatial division scheme^8^. The LP filters (P_1_, P_2_, P_3_, and P_4_) are simply nanowire gratings oriented in four different orientations, 0, 90, −45, and 45°, with respect to the *x*-axis (Fig. 5a). The two CP filters, P_5_ and P_6_, are selectively transmitting only LCP and RCP light, respectively (${\mathrm{P}}_5^\prime$ and ${\mathrm{P}}_6^\prime$ are backup structures for P_5_ and P_6_). An empty cell P_0_ without any filters is located in the center to measure the total light intensity I_0_. All the filters and the empty cells have the same area of 80 µm × 80 µm. The incident light is filtered with these six spatially distributed micrometer-size polarization filters and then measured by separately photodetectors to obtain the polarized components. Given the light transmitted by P_*i*_, denoted I_*i*_ (*i* = 0–6), the Stokes parameters, (S_0_, S_1_, S_2_, and S_3_), of the incident light are then calculated as follows:^8^

$$\left\{ {\begin{array}{*{20}{l}} {S_0 = I_0} \\ {S_1 = I_2 - I_1} \\ {S_2 = I_4 - I_3} \\ {S_3 = I_6 - I_5} \end{array}} \right.$$

Notably, this design can be used to detect the SoP of any incident light, including partially polarized light. When the incident light is fully polarized, I_0_^2^ = S_1_^2^ + S_2_^2^ + S_3_^2^, while for partially polarized light, I_0_^2^ > S_1_^2^ + S_2_^2^ + S_3_^2^.

To test the performance of the microscale polarization filter array fabricated, we used FTIR with an infrared microscope to build the measurement setup, as shown in Fig. 5a. Unpolarized light from the FTIR internal source was transmitted through a conventional linear polarizer and tuneable LC waveplate, separated by a reflective objective (also known as a condenser), to generate an arbitrary SoP. The reflective objective was used to focus the light onto the device, which was placed on a motorized stage. By placing the tuneable LC waveplate between the objective and the device under testing, we were able to minimize the impact of the reflective objective on the SoP of the light incident on the device. The light transmitted through the sample was then collected by an infrared objective. An aperture was used at the image plane to select the measurement region (30 × 30 µm^2^) on the device. Such a scanning imaging system allows us to characterize the light transmitted through all seven cells by simply changing the lateral displacement of the motorized stage. For calibration purposes, we removed the sample and used a rotating PA to characterize the SoP of the incident light (see “Materials and methods” section for more details). Figure 5b presents the polar plots for four representative SoPs. The measured results obtained with the on-chip polarimeter (red) are consistent with those obtained from the PA (black). We extracted the ellipse plots for all the SoPs to evaluate the accuracy of the orientation and ellipticity angle measurements. Based on all the measurement results of eight SoPs (see “results” for the other SoPs in Supplementary Information, Fig. S11), the average errors of the orientation and ellipticity angles are 0.046° and 2.21°, respectively. Moreover, the handedness of the light can be obtained with our technique, as shown on the ellipse plots labeled with blue arrows, which is not available from the measurements taken by the rotating polarizer. Figure 5c shows a summary of the Stokes parameters (S_0_, S_1_, S_2_, and S_3_) obtained by our device (all the Stokes parameters were normalized by S_0_). To evaluate the accuracy of the measurements, we characterized each SoP with the rotating PA and the extracted corresponding Stokes parameters (shown in Fig. 5c). The average measurement errors of S_1_, S_2_, and S_3_ were 1.9%, 2.7%, and 7.2%, respectively. Based on the Stokes parameters measured, the measurement errors for the degree of LP ($\sqrt {S_1^2 + S_2^2} /S_0$) and degree of circular polarization (*S*_3_/*S*_0_) are 3.3% and 7.9%, respectively. Compared with other plasmonic or dielectric metasurface-based polarimetric detection techniques reported in the literature, our devices exhibit the best performances in measurement accuracy and optical efficiency (Table 1). Even though we used a scanning imaging system to perform the device characterization in our experiment, a spatial division measurement scheme can be employed to perform full-Stokes polarization detection in one snapshot without moving parts. In such a configuration, the polarization filter array is directly integrated on the top of a photodetector array so that the incident light filtered by each microscale polarization filter is measured by the corresponding photodetector beneath that filter. Thus, all the polarization components can be obtained by a photodetector array integrated with microscale polarization filters.

## Discussion

Here, we reported bioinspired chiral double-layer metasurface structures (ODLMs) with both strong chiral optical effects and high optical efficiencies. Based on these structures, we have experimentally demonstrated submicron-thick CP light filters with CPER as high as 35 and maximum transmission efficiency close to 80%. The best operational wavelengths can be tailored by varying the design parameters from 1.3 to 1.6 µm. We attribute the high transmission efficiency and extinction ratio to the low insertion loss of the dielectric metasurface QWP and the nanowire linear polarizer, the high accuracy of the phase delay of the metasurface QWP, and the high LPER of the nanowire linear polarizer. The proposed ODLM-based CP filers can be very compact, with thickness less than 1 µm and minimal lateral dimensions of 1 × 1 µm^2^ (to maintain a CPER greater than 20 and transmission efficiency > 60%; see Fig. S13). Our experimental and theoretical analyses show that the device performance could be further enhanced by improving the fabrication procedures and improving the performance and bandwidth of the metasurface QWP^49^. We also monolithically integrated the ODLM-based CP filers with LP nanograting filters for full-Stokes polarization measurements of input light with arbitrary polarization states. Our polarimetric detection devices demonstrate the best performance in measurement accuracy and optical efficiency (80%). In addition to their superior performance, the chiral metasurface structures and polarimetric devices presented exhibit various advantages, such as easy on-chip integration, ultracompact footprint, manufacturing scalability, and broad wavelength range. Therefore, our work holds great promise for chip-integrated systems for quantum-based optical computing and information processing, CD spectroscopy, polarimetric imaging, and sensing applications.

## Materials and methods

### Numerical simulations

The FDTD simulations were performed using Lumerical Inc. FDTD solver. The material optical properties of the *α*-Si and silicon oxide were determined from ellipsometry measurements using a J.A. WOOLLAM system. The residual SiO_*x*_ mask layer and sidewall tilting were considered in accordance with the SEM results. Here, we simulated one unit cell with normal plane wave source(s) incidence, the in-plane boundary conditions were periodic, and perfectly matched layers were used for the out-of-plane boundaries. CP light was simulated by a superposition of two linearly polarized sources with *π*/2 relative phase retardance. We set a refined mesh with a minimum mesh size of 5 nm for silicon nanopillars. We confirmed that all the simulations converged at an auto-shutoff value of 10^–5^.

### Fabrication

1. Gold polarizing nanograting: The fused silica substrate was spin coated with double-layer poly(methyl methacrylate) (PMMA) (170 nm, 495 k followed with 35 nm, 950 k) and a very thin (~5 nm) thermally evaporated Cr layer for charge dissipation. Then, the samples were exposed by ebeam lithography (EBL, JEOL JBX-6000FS) and developed in a mixture of methyl isobutyl ketone (MIBK) and isopropanol (IPA) at a ratio 1:3. The sample was cleaned by oxygen plasma (PlasmaTherm 790, 5 sccm O2 with 8 mTorr chamber pressure, 20 W) for a few seconds to remove the residual PMMA on the exposed region. Then, a 4 nm adhesive Cr layer followed by 122 nm of gold was deposited by thermal evaporation (Edwards Auto 306) without breaking vacuum. Subsequently, the Au/Cr nanogratings were lifted off by soaking the sample in acetone for more than 12 h followed by sonication for 1 min.
2. Spacer deposition: A 5-min oxygen plasma etch was applied to the samples to completely remove organic contaminants. Then, the samples were immediately placed in the sputtering chamber (Lesker PVD 75) and covered with a 350 nm SiO_*x*_ spacer layer (RF power 250 W) at a rate of 0.6 Å/s.
3. Si QWP fabrication: Amorphous silicon (α-Si) of 700 nm was deposited by PECVD (Oxford Plasmalab 100, 350 °C/15 W) on the SiO_*x*_ spacer layer, followed by deposition of 160 nm SiO_*x*_ (350 °C/20 W) without breaking vacuum as a hard mask layer. Another 10 nm Cr layer was deposited onto the sample surface to avoid charging effects during EBL fabrication. Again, a double-layer PMMA resist (120 nm, 495 k followed with 50 nm, 950 k) was coated on the sample, exposed with EBL, and developed in IPA/MIBK as described earlier. Then, 60 nm of Cr was thermally evaporated onto the sample and lifted off in acetone as described earlier to form the Cr hard mask. Next, the 10 nm Cr adhesion layer was etched through by ICP etching (Advanced Vacuum Apex SLR, Cl_2_/O_2_ 22/8 sccm, 20 mTorr, ICP/ bias power 400/100 W) to form isolated Cr nanostructure masks, which further masked the anisotropic etching of the SiO_*x*_ hard mask by RIE (Plasmatherm RIE 790, CHF_3_/ O_2_ 40/3 sccm, 40 mTorr, 250 W) and stopped at the α-Si layer. Then, the Cr was removed by CR-4s (Cyantek) etchant, and the α-Si layer was etched by ICP RIE (ICP/bias power of 250/140 W, 10 mTorr, Cl_2_:Ar = 100/5 sccm) using the SiO_*x*_ mask to complete the device fabrication.

### Measurements

The optical transmission measurements were performed using a Bruker Vertex 70 FTIR spectrometer connected to a Hyperion 2000 mid-IR microscope (Fig. S10). For the QWP measurements, we used one linear polarizer in the optical path immediately in front of the sample under testing to ensure LP incidence. Another linear polarizer was placed in the optical path immediately behind the sample to analyze the polarization state of the output light by rotating the polarizer and measuring the transmitted light with an IR photodetector. Two 15× objective and condenser lenses with N.A. = 0.4 were used. For CP detection measurements and full-Stokes parameter measurements, we used one linear polarizer and an LC waveplate (LCC1223-C by Thorlabs, Inc.) to generate CP light with both handedness and arbitrary polarization state that was incident to the device under testing. As a calibration process, we characterized the polarization state of the incident light with a rotating linear polarizer and QWP. All the transmission spectra were normalized with respect to that of the bare fused silica substrate to eliminate the impact of the substrate.

## Supplementary information

Supplementary Information.
