# Supplementary material for: Nature-inspired chiral metasurfaces for circular polarization detection and full-Stokes polarimetric measurements
Source: Light Sci Appl. 2019 Aug 28;8:78. doi: 10.1038/s41377-019-0184-4 (PMC6804686; doi:10.1038/s41377-019-0184-4)
Supplement: Supplementary file 1 — Supplementary Information. [file 41377_2019_184_MOESM1_ESM.docx]

Supplementary Information for:

**“Nature-Inspired Chiral Metasurfaces for Circular Polarization Detection and Full-Stokes Polarimetric Measurements”**

*Ali Basiri*^1,2^*, Xiahui Chen*^1,2^*, Jing Bai*^1,2^*, Pouya Amrollahi^3^, Joe Carpenter*^1,2^*, Zachary Holman*^1,2^*, Chao Wang*^1,2,3*^*, Yu Yao*^1,2*^

^1^School of Electrical, Computer and Energy Engineering, Arizona State University, Tempe, AZ, USA, 85281

^2^Center for Photonic Innovation, Arizona State University, Tempe, AZ, USA, 85281

^3^Biodesign Center for Molecular Design & Biomimetics, Arizona State University, Tempe, AZ, USA, 85281

***Corresponding authors：** [**yuyao@asu.edu**](mailto:yuyao@asu.edu)**,** [**wangch@asu.edu**](mailto:wangch@asu.edu)

Contents

[1. A simplified model of the proposed ODLM based on Jones matrices 2](#_Toc13063933)

[2. Extraction of electric field components from angle-dependent transmission measurements 2](#_Toc13063934)

[3. Design of the ODLM chiral metamaterial structures 2](#_Toc13063935)

[4. Fabrication of the ODLM chiral metamaterial structures 2](#_Toc13063936)

[5. Evaluation of ODLM structures tolerance to imperfections during fabrication process 4](#_Toc13063937)

[6. Measurement setup and full-Stokes polarimetry results 7](#_Toc13063938)

[7. Measurement results for a pair of complementary RCP and LCP detection devices 9](#_Toc13063939)

[8. Comparison of chip-integrated CP filters 9](#_Toc13063940)

[9. Dependence of device performance on pixel size 9](#_Toc13063941)

[10. On-chip integration of full-Stokes polarimetric device 9](#_Toc13063942)

[11. Supplementary references 9](#_Toc13063943)

## A simplified model of the proposed ODLM based on Jones matrices

To reveal the major design criteria, we first set up a simplified model of the proposed ODLM structure based on Jones matrices:

$$M_{ODLM}=M_{NGLP}\boldsymbol{\times}M_{QWP}$$

Where:

$$M_{QWP}=e^{-i\frac{\Delta\varphi}{2}}\left( \begin{matrix} {\gamma_{f}\cos}^{2} \theta+{e^{i\Delta\varphi}\gamma_{s}\sin}^{2} \theta& \left( \gamma_{f}-e^{i\Delta\varphi}\gamma_{s} \right)\cos\theta\sin\theta\\ \left( \gamma_{f}-e^{i\Delta\varphi}\gamma_{s} \right)\cos\theta\sin\theta& {\gamma_{f}\sin}^{2} \theta+{e^{i\Delta\varphi}\gamma_{s}\cos}^{2} \theta\end{matrix} \right)$$

$$M_{NGLP}=\left( \begin{matrix} \gamma_{x}^{V/H} & 0 \\ 0 & \gamma_{y}^{V/H} \end{matrix} \right)$$

And therefore:

$$M_{ODLM}=e^{-i\frac{\Delta\varphi}{2}}\left( \begin{matrix} \gamma_{x}^{V/H}\left( {\gamma_{f}cos}^{2} \theta+{e^{i\Delta\varphi}\gamma_{s}sin}^{2} \theta\right) & \gamma_{x}^{V/H}\left( \gamma_{f}-e^{i\Delta\varphi}\gamma_{s} \right)\cos\theta\sin\theta\\ \gamma_{y}^{V/H}\left( \gamma_{f}-e^{i\Delta\varphi}\gamma_{s} \right)\cos\theta\sin\theta& \gamma_{y}^{V/H}\left( {\gamma_{f}sin}^{2} \theta+{e^{i\Delta\varphi}\gamma_{s}cos}^{2} \theta\right) \end{matrix} \right)$$

Where $\Delta\varphi=\varphi_{y}-\varphi_{x}=-\frac{\pi}{2}+{\Delta\varphi}^{var}$ is the phase retardation difference between fast and slow axes introduced by the QWP (note its fast axis is oriented at angle $\theta$ with respect to x-axis) and $\gamma_{f}$ and $\gamma_{s}$ are transmission coefficients for electric field components along the fast and slow axes, respectively. $\gamma_{x}^{V/H}$and $\gamma_{y}^{V/H}$ are transmission coefficients of the nanograting linear polarizer (NGLP), oriented horizontally (H) or vertically (V), for electric field components along x and y axes. For simplification, this heuristic model ignores multiple reflection between the silicon metasurface and the NGLP.

For the LCP-ODLM design, which selects LCP light over RCP light (left panel in Figure 1b, with $=-\frac{\pi}{2}$ ), the extinction ratio is:

$${CPER}_{LCP}=\frac{\left| \gamma_{x}^{V/H}\gamma_{f}e^{-i(\frac{\Delta\varphi}{2}+\frac{\pi}{4})}+\gamma_{x}^{V/H}\gamma_{s}e^{i(\frac{\Delta\varphi}{2}+\frac{\pi}{4})} \right|^{2}+\left| {-\gamma}_{y}^{V/H}\gamma_{f}e^{-i(\frac{\Delta\varphi}{2}+\frac{\pi}{4})}+\gamma_{y}^{V/H}\gamma_{s}e^{i(\frac{\Delta\varphi}{2}+\frac{\pi}{4})} \right|^{2}}{\left| \gamma_{x}^{V/H}\gamma_{f}e^{i(-\frac{\Delta\varphi}{2}+\frac{\pi}{4})}+\gamma_{x}^{V/H}\gamma_{s}e^{-i(-\frac{\Delta\varphi}{2}+\frac{\pi}{4})} \right|^{2}+\left| {-\gamma}_{y}^{V/H}\gamma_{f}e^{i(-\frac{\Delta\varphi}{2}+\frac{\pi}{4})}+\gamma_{y}^{V/H}\gamma_{s}e^{-i(-\frac{\Delta\varphi}{2}+\frac{\pi}{4})} \right|^{2}}$$

As a simplified illustration, in Figure 1b on the left panel, we can consider ideal QWP, i.e. $\Delta\varphi$=$-\frac{\pi}{2}$ and $\gamma_{f}=\gamma_{s}=1$, as well as ideal horizontal nanograting with $\gamma_{y}^{V/H}=1$and$\gamma_{x}^{V/H}=0$. Hence, the equation above boils down to the following expressions for LCP and RCP incident light:

$${M_{tot}I_{LCP}=M}_{tot}\frac{1}{\sqrt{2}}\left( \begin{aligned} 1 \\ i \end{aligned} \right)=e^{i\frac{\pi}{2}}\left( \begin{aligned} 0 \\ 1 \end{aligned} \right)$$

$${M_{tot}I_{RCP}=M}_{tot}\frac{1}{\sqrt{2}}\left( \begin{aligned} 1 \\ -i \end{aligned} \right)=\left( \begin{aligned} 0 \\ 0 \end{aligned} \right)$$

Which basically means transmitting the LCP incidence and blocking the RCP one, confirming our intuitive understanding of the structure, as well as the FDTD simulations.

## Extraction of electric field components from angle-dependent transmission measurements

The phase difference and amplitude of x and y components of the electric field in Figure 4a, as well as the discrete data points in polarization ellipse plots in Figure 5b are extracted from angle-dependent transmission measurements using a rotating polarization analyser (PA) based on the following set of equations:

$T_{\theta_{1}}=\left| \left| E_{x} \right|\cos\theta_{1}+\left| E_{y} \right|e^{i\left( \varphi_{y}-\varphi_{x} \right)}\sin\theta_{1} \right|^{2}$=$\left( \left| E_{x} \right|\cos\theta_{1}+\left| E_{y} \right|e^{i\left( \varphi_{y}-\varphi_{x} \right)}\sin\theta_{1} \right)\left( \left| E_{x} \right|\cos\theta_{1}+\left| E_{y} \right|e^{-i\left( \varphi_{y}-\varphi_{x} \right)}\sin\theta_{1} \right)=\left| E_{x} \right|^{2}\cos^{2} \theta_{1}+\left| E_{y} \right|^{2}\sin^{2} \theta_{1}+2\left| E_{x} \right|\cos\theta_{1}\left| E_{y} \right|\sin\theta_{1}\cos\left( \varphi_{x}-\varphi_{y} \right)$

$$T_{\theta_{2}}=\left| \left| E_{x} \right|\cos\theta_{2}+\left| E_{y} \right|e^{i\left( \varphi_{y}-\varphi_{x} \right)}\sin\theta_{2} \right|^{2}$$

$$T_{\theta_{3}}=\left| \left| E_{x} \right|\cos\theta_{3}+\left| E_{y} \right|e^{i\left( \varphi_{y}-\varphi_{x} \right)}\sin\theta_{3} \right|^{2}$$

Where$\theta_{1}$, $\theta_{2}$ and $\theta_{3}$ are three arbitrary angles for PA. By solving this system of equations, we can retrieve the values corresponding to$E_{x}$, $E_{y}$ and$\left| \varphi_{x}-\varphi_{y} \right|$.

## Design of the ODLM chiral metamaterial structures


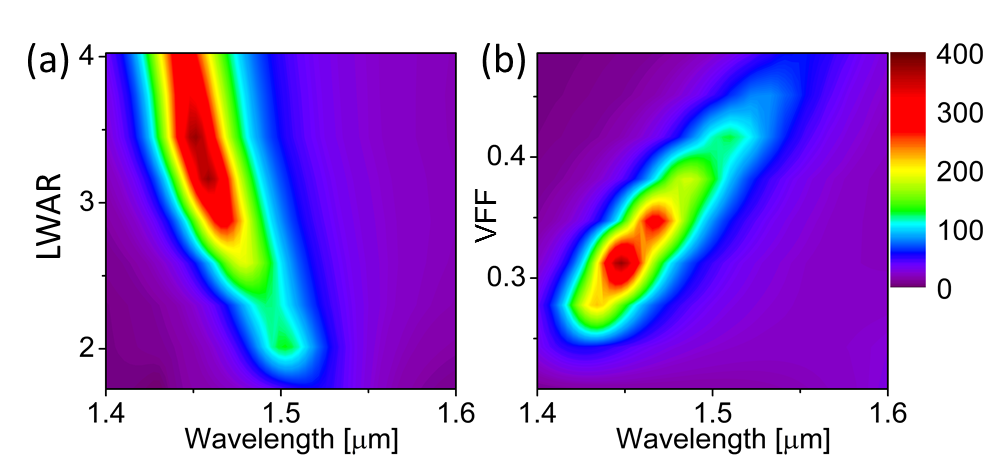


**Figure S1. Wavelength tunability and bandwidth of ODLM structure.** (a) Scanning of the length-to-width aspect ratio and (b) volume filling factor of the dielectric metasurface on top of the grating with 350 nm SiOx spacer layer, demonstrating the tunability of operation wavelength and bandwidth. In our simulations, we have considered 100 nm SiOx mask layer residue on the a-Si surface, as well as 5 degrees tilting angle for the QWP side walls obtained by SEM imaging after ICP etching.


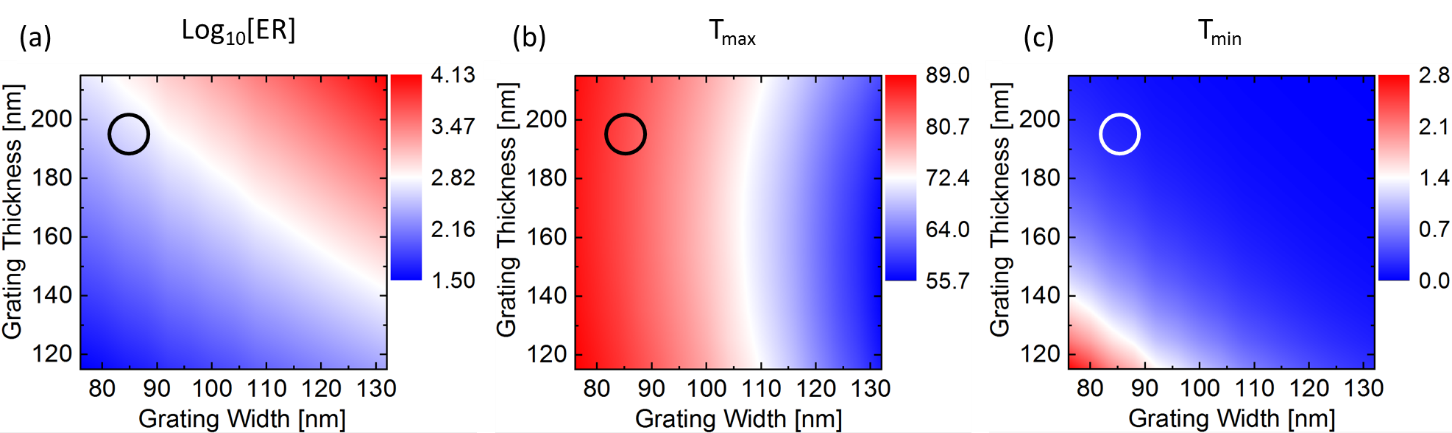


**Figure S2. Nanograting extinction ratio and transmission.** (a) Optimization of nanograting extinction ratio (shown in logarithmic scale) by sweeping its thickness and width, while keeping the periodicity half of the QWP array (230 nm). (b) Maximum and (c) minimum transmission through nanograting corresponding to perpendicular and parallel axes with respect to the nanograting orientation, respectively. The marked circles indicate the chosen point of reasonable balance (corresponding to width=84 nm and thickness=195 nm) between extinction ratio and grating transmission, and hence used for ODLM device simulations.

## Fabrication of the ODLM chiral metamaterial structures


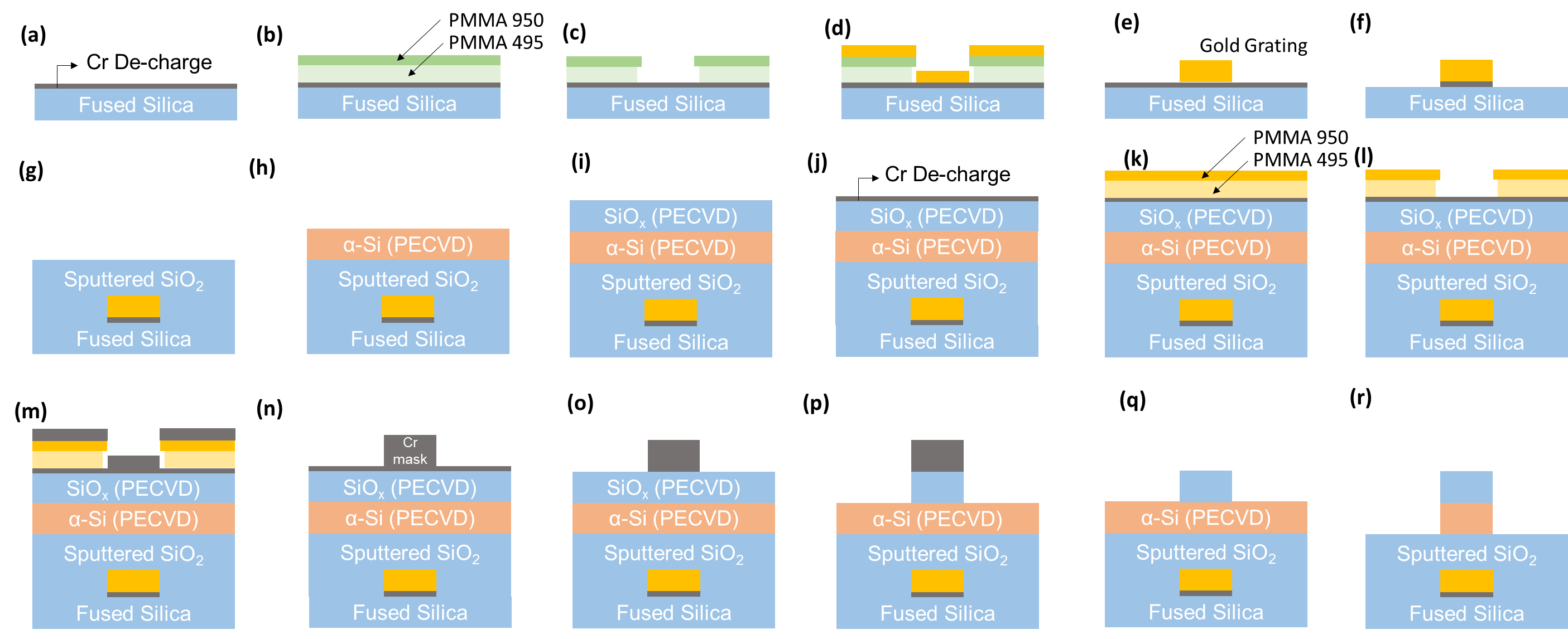


**Figure S3. Fabrication steps of nanograting and silicon metasurface.** (a-f) Nanograting fabrication performed by electron-beam lithography, thermal evaporation of gold and lift-off. (g-r) silicon nanopillars fabrication.


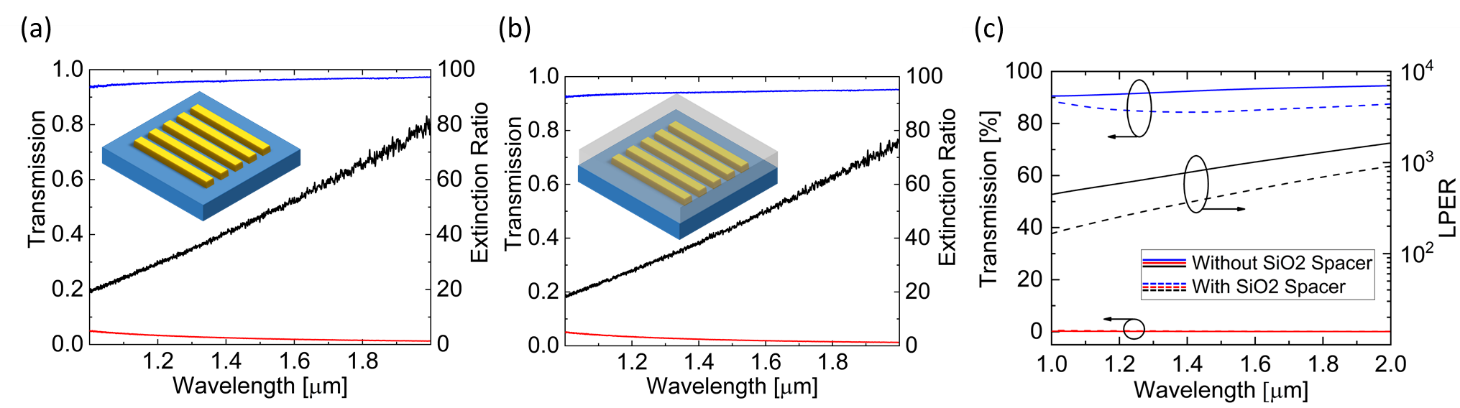


**Figure S4. Measured nanograting efficiency and extinction ratio.** (a) Before deposition of spacer layer. (b) After deposition of 350 nm spacer layer. (c) Simulation results for comparison. The black and red curves represent the maximum and minimum transmission perpendicular and along the nanograting, respectively, and blue curves show the calculated LPER from the ratio of the transmissions (no background noise reduction is considered in measurement results here).

## Evaluation of ODLM structures tolerance to imperfections during fabrication process


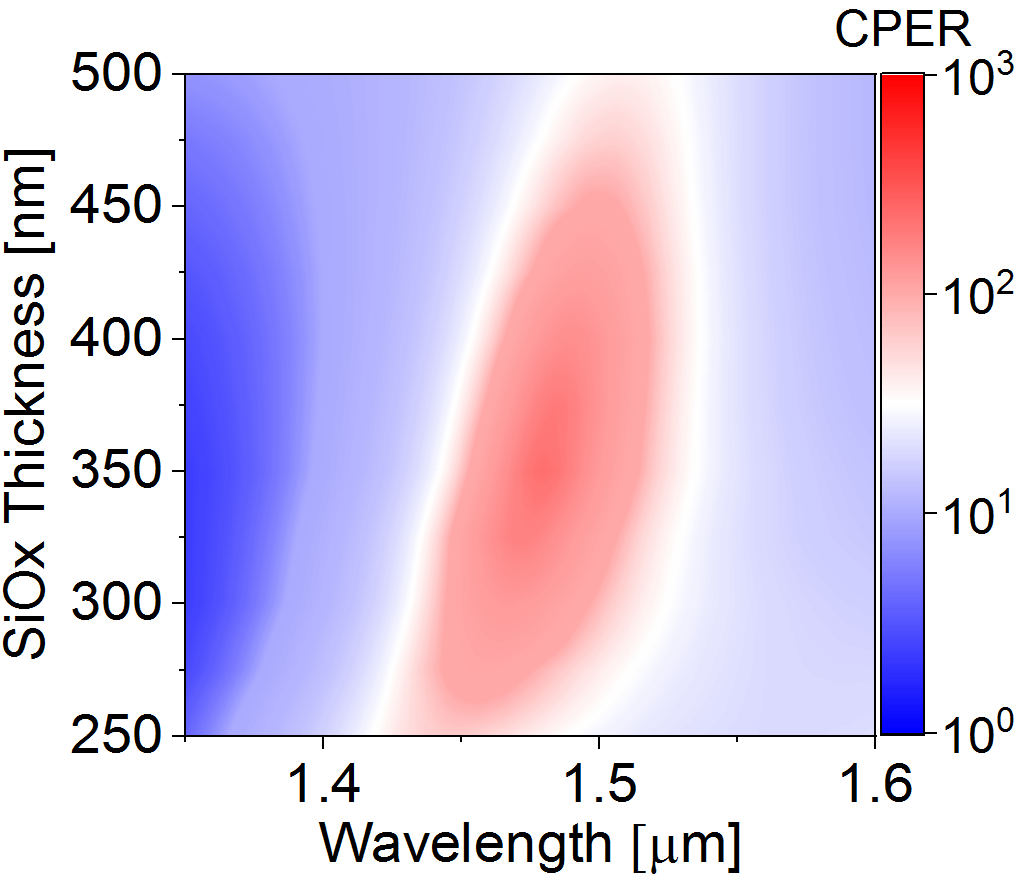


**Figure S5. SiO_x_ spacer layer optimization.** Simulated CPER using FDTD method, with various SiO_x_ spacer layer thicknesses, ranging from 250 to 500 nm.


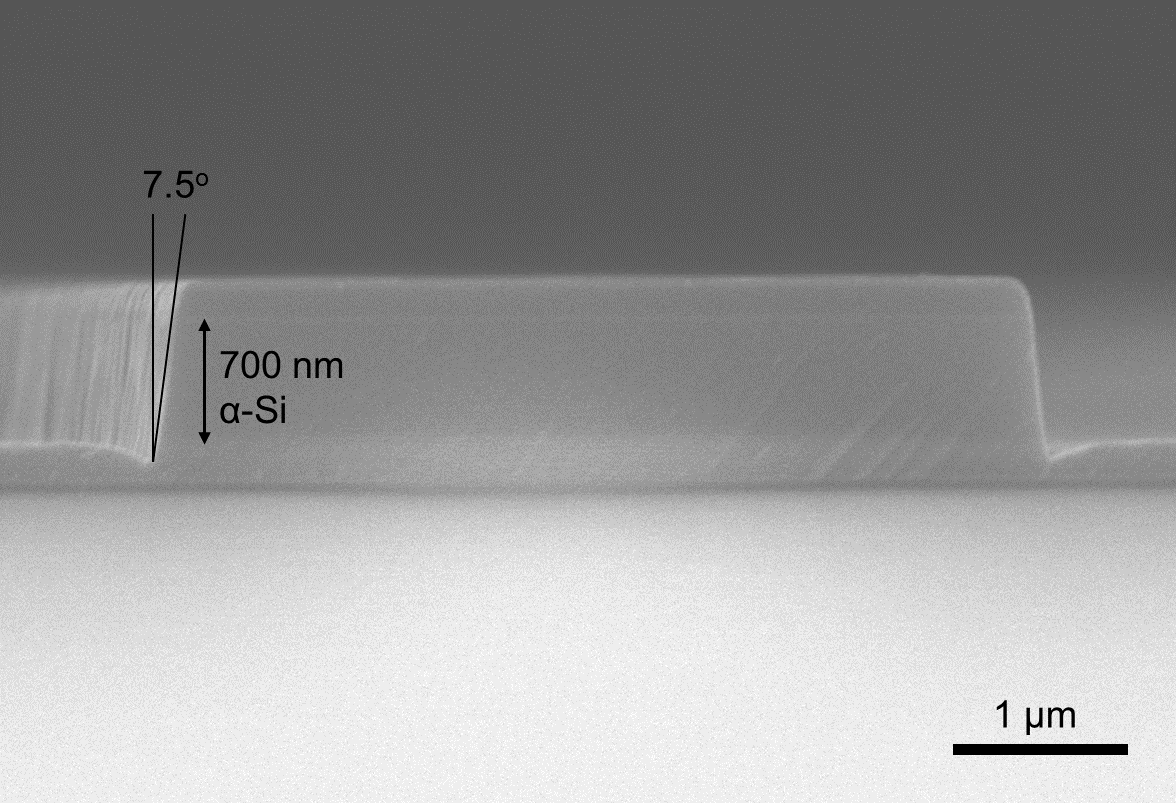


**Figure S6. Sidewall tilting angle after silicon etching.** The SEM image showing around 7.5 degrees slope on the sidewall of silicon nanopillars. The top thinner layer shows the residue of SiO_x_ hard mask after ICP etching of silicon metasurface.


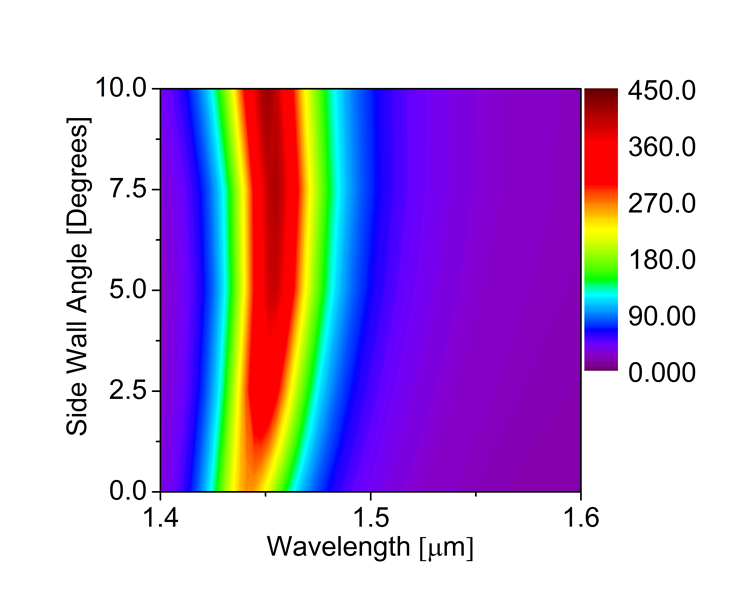


**Figure S7. CPER dependence on side-wall tilting angle.** Simulated results of extinction ratio of ODLM device as a function of side-wall tilting angle of silicon nanopillars, caused by variation of silicon etching conditions during the fabrication process. This result indicates that slight variation of side-wall angle does not contribute significantly to the device performance. Nevertheless, its average value can be taken into consideration prior to the fabrication to avoid large deviation from expected working wavelength and overall extinction ratio.


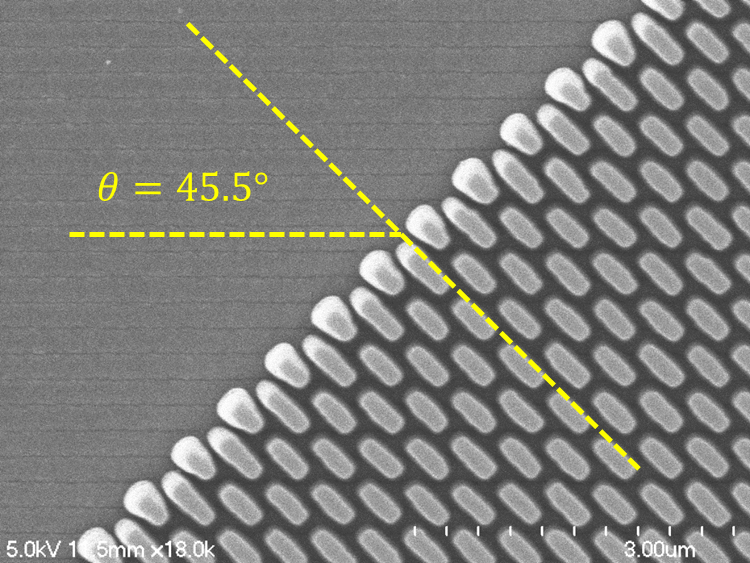


**Figure S8. Alignment of silicon nanopillars on top of gold nanograting.** The relative rotation angle of top and bottom layers after alignment is measured using SEM imaging, indicating less than $0.5^{\circ}$ fabrication inaccuracy.


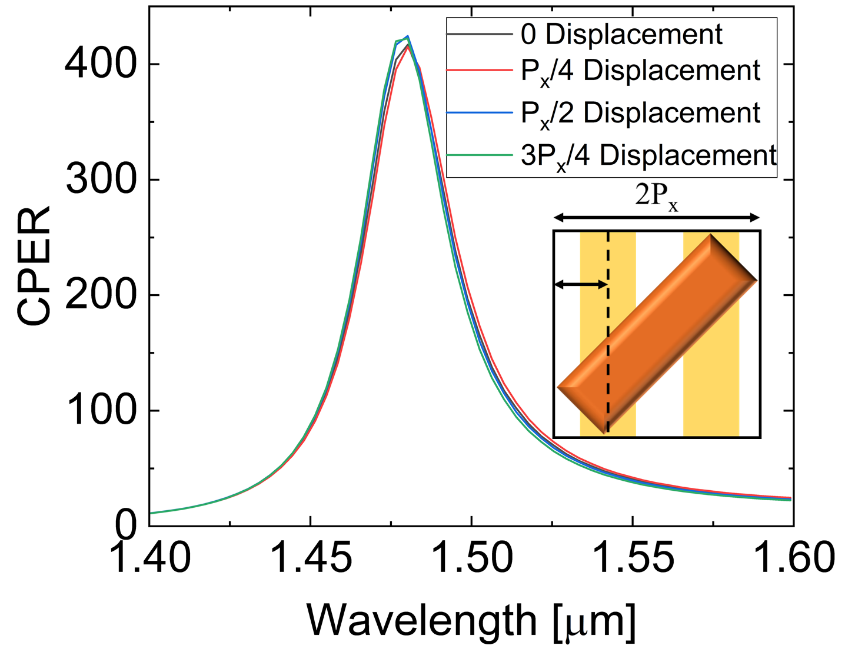


**Figure S9. Nanograting misplacement simulation.** FDTD simulation results indicating that lateral displacement of nanograting with respect to QWP only affects the CPER figure of merit by less than 2 %.

## Measurement setup and full-Stokes polarimetry results


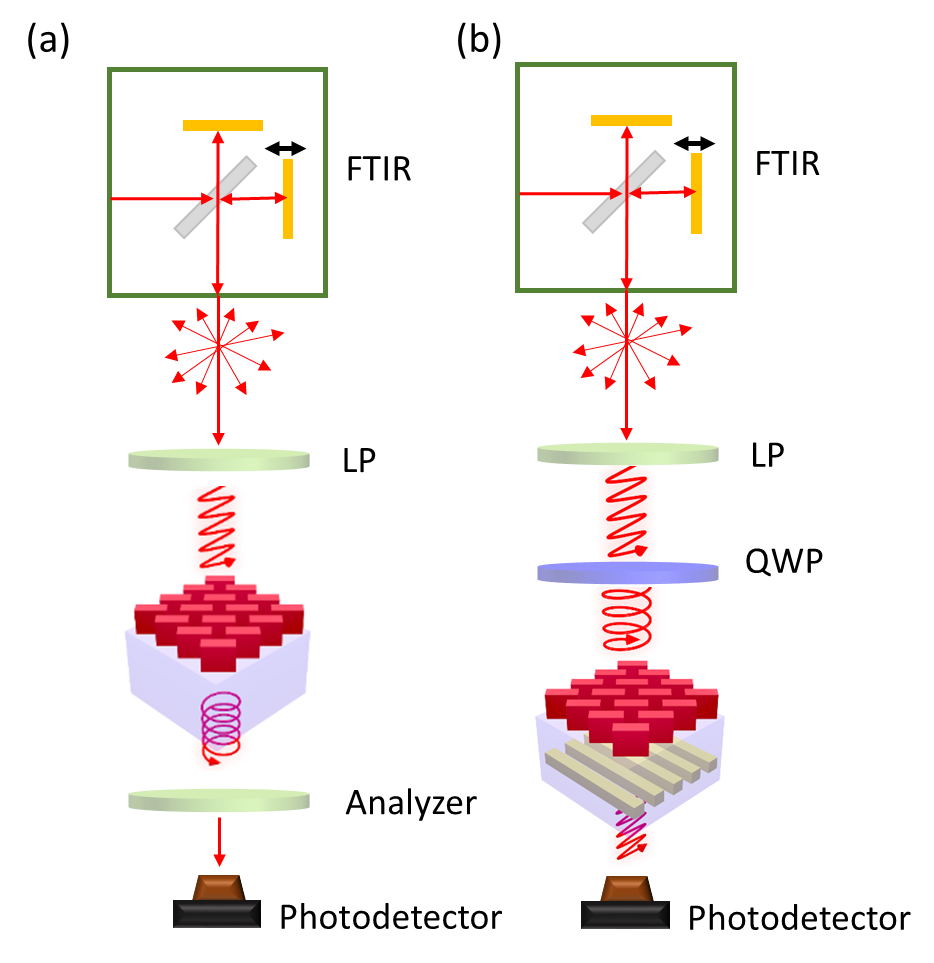


**Figure S10. Device characterization setup.** (a) QWP performance characterization using FTIR. The incident unpolarized light from the NIR source in FTIR is converted to linearly polarized light using a conventional linear polarizer (LP) (b) ODLM device performance characterization by generating CP incident light via superposition of a LP and liquid crystal quarter waveplate (QWP).


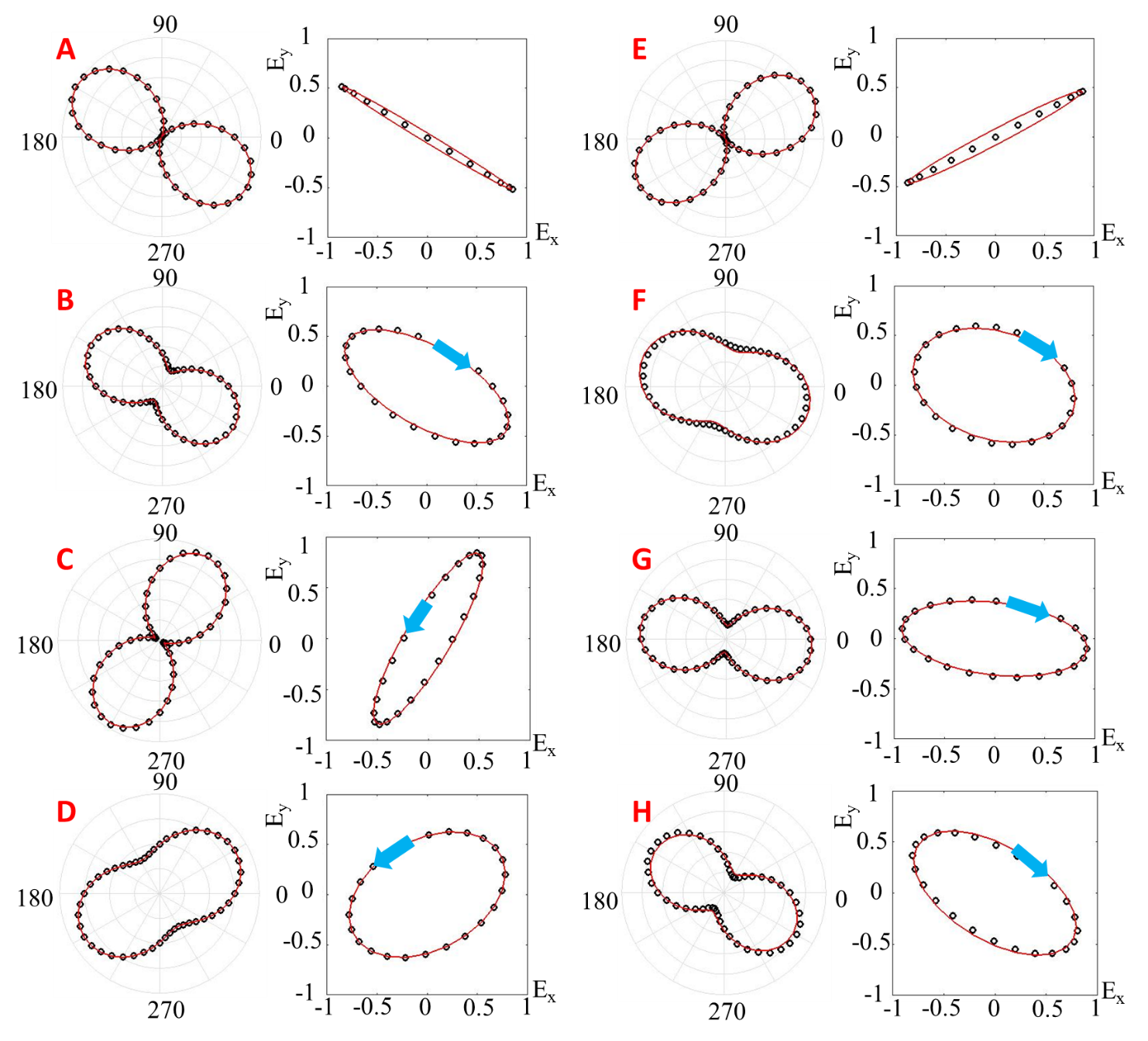


**Figure S11. Complete determination of polarization information of arbitrary incident light.** Comparison of polar plots (left panels) and polarization ellipse plots (right panels), obtained by our sample and a conventional linear polarizer analyzer, corresponding to eight different arbitrarily polarized incident light inputs. The blue arrows indicate the handedness of polarization of incident light, only achievable by our device.

## Measurement results for a pair of complementary RCP and LCP detection devices


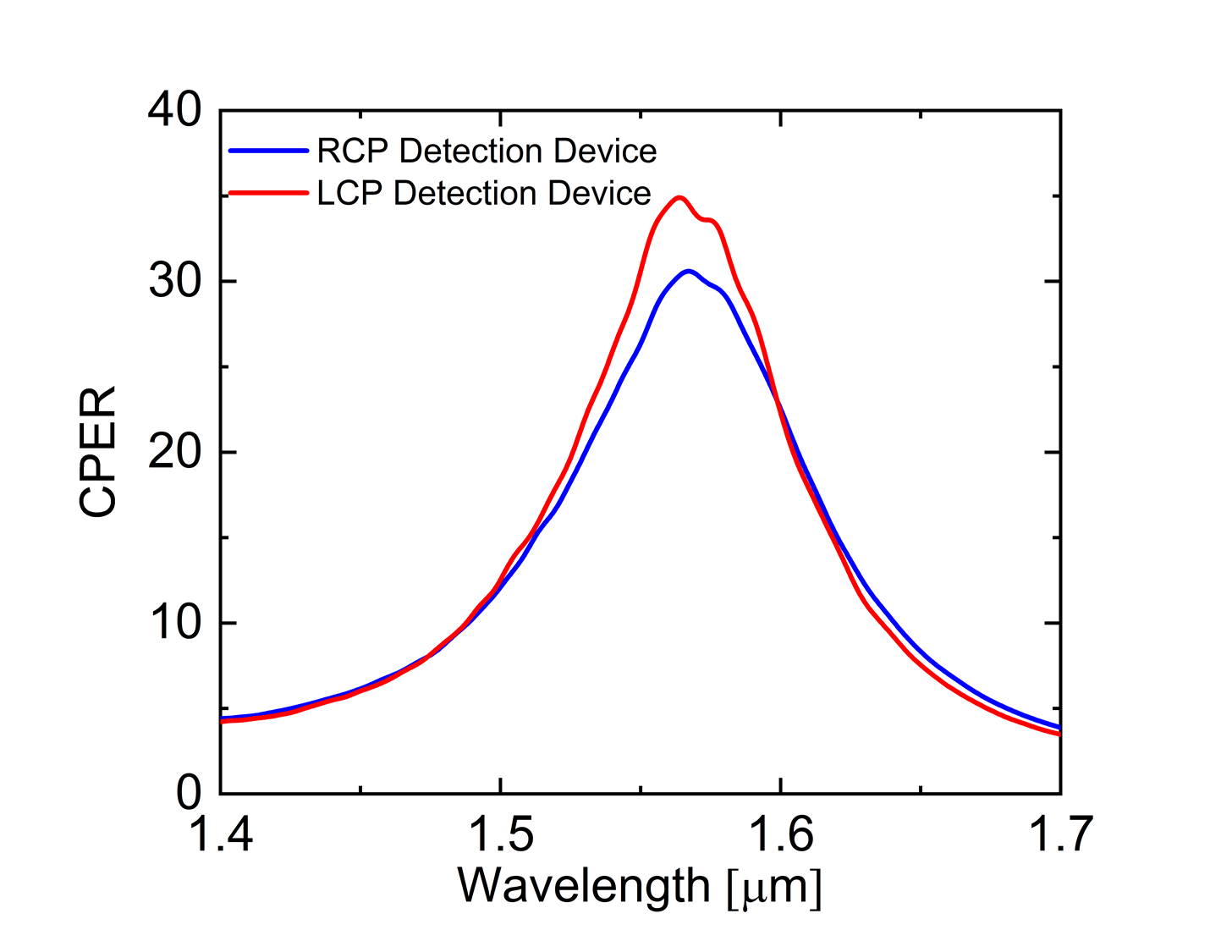


**Figure S12. CPER comparison of RCP and LCP detection devices.** RCP and LCP detection devices with their QWP layers rotated 90 degrees with respect to each other. Both devices are designed for the same wavelength range and fabricated side by side on a single chip to selectively pass the desired handedness of incident CP light.

## Comparison of chip-integrated CP filters

**Table S1.** Solid-state-based solutions for CPL filters.

| Artificial Chiral Materials | 3D metallic structures (helical^1^, spiral^2^, L-shaped^3^, etc.) | Chiral plasmonic metasurfaces(spiral^4^, gammadion^5^, hot electron injection^6^) | Stacked multilayer structures (crosses^7^, nanorods^8,9^) | Dielectric chiro-optical gammadion nanostructure^10^ | Dielectric 2D chiral metasurface^11^ | This work (ODLM-based metasurface) |
| --- | --- | --- | --- | --- | --- | --- |
| Measured CPER | ~ 15 (^1^),  NA (^2^),  < 1.2 (^3^) | > 50 (^4^),  < 1.5 (^5^),  ~ 3.4 (^6^) | NA (^7^),  ~2 (^8^),  < 5 (^9^) | ~8 | < 8 | ~ 35 |
| Operational wavelength (nm) | 3.5-6.5 μm (^1^),  0.75-3 μm (^2^),  4-6 μm (^3^) | ~0.8$\mu m$ (^4^),  ~0.8-1$\mu m$(^5^),  1.2-1.5 $\mu m$(^6^) | ~1.55 $\mu m$ (^7^),  0.9-1.1$\mu m$ (^8^),  0.75-1.05$\mu m$ (^9^) | 540 nm | 1.5-1.67 $\mu m$ | 1.3-1.6 |
| 3dB Bandwidth (nm)* | ~3 μm (^1^),  < 400 nm (^2^),  ~500 nm (^3^) | NA (^4^),  ~150 nm (^5^),  ~200 nm (^6^) | NA (^7^),  ~ 200 nm (^8^),  < 400 nm (^9^) | ~ 30 nm | < 100 nm | 100-200 nm |
| Operation mode | Transmission | Absorption, photocurrent | Transmission | Transmission | Transmission | Transmission |
| Efficiency | ~ 80 % (^1^),  ~ 80 % (^2^),  < 85 % (^3^) | NA (^4^),  < 35 % (^5^),  NA (^6^) | NA (^7^),  < 60 % (^8^),  < 70 % (^9^) | ~ 90 % | ~ 80 % | ~ 80 % |
| Manufacturing scalability | Challenging (complex 3D fabrication) | Yes | Yes (sensitive to alignment between multilayers) | Yes (Requiring precise nanostructure patterning) | Yes (Requiring precise nanostructure patterning) | Yes |

* The bandwidth is the wavelength range where CPER is larger than half of its peak value.

## Dependence of device performance on pixel size

Figure S13 shows the FDTD simulation results for dependence of CP filters on pixel dimensions. Here we have used PML boundary conditions for all directions. Then considered square-shaped gold frames with 530 nm width and the same height and thickness as nanogratings to enclose them and eliminate diffraction from PML edges. As shown below, a CPER close to 20 with more than 60 % transmission can be achieved by a minimum pixel size of $1\times1 \mu m^{2}$.


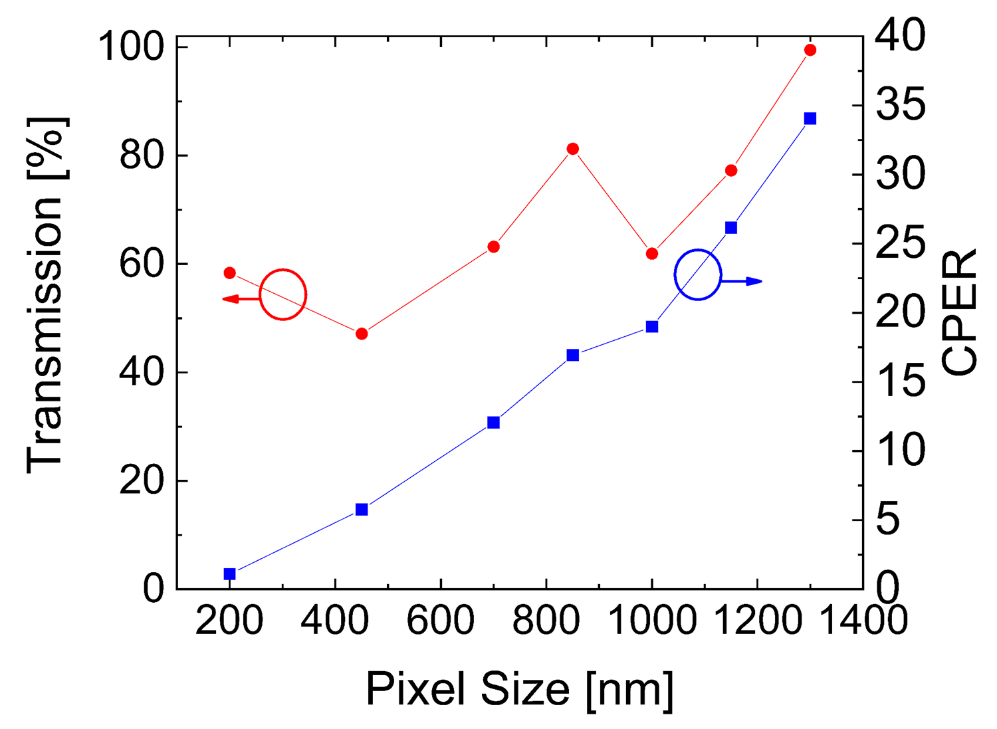


**Figure S13. Simulation results for transmission and CPER dependence on pixel size.** Simulated transmission (left axis) and CPER (right axis) as a function of square-shaped pixel size. Based on these results, a CPER close to 20 with more than 60 % transmission can be achieved by a minimum pixel size of $1\times1 \mu m^{2}$.

## On-chip integration of full-Stokes polarimetric device

Figure S14 depicts an array of devices with 3 by 3 super-pixels for full-Stokes polarimetric imaging, corresponding to Figure 5a in the main text. The optical microscope image in center shows the overall layout of $80\times80 {\mu m}^{2}$units. The SEM images correspond to each individual unit, respectively.


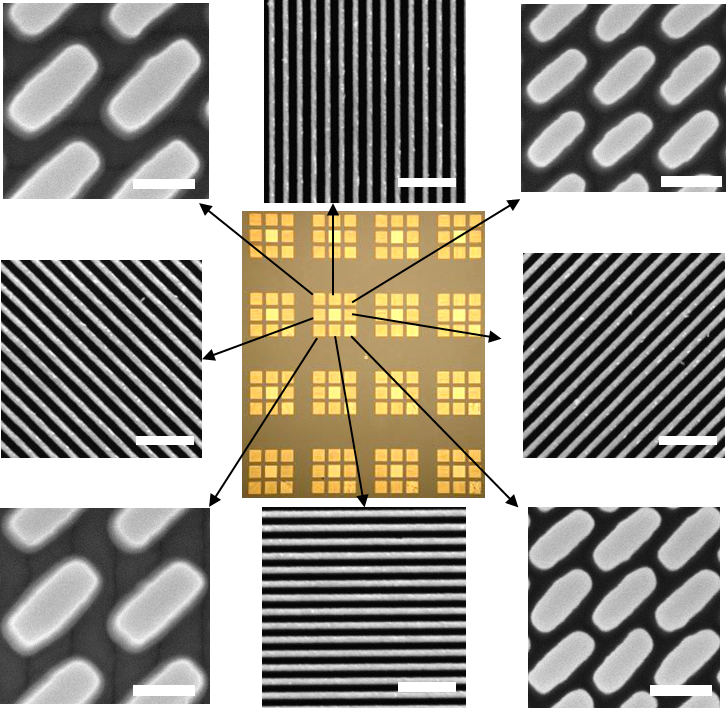


**Figure S14. On-chip integration for full-Stokes polarimetry imaging.** Optical image (center), as well as SEM images of different Stokes measurement units ($80\times80 {\mu m}^{2}$) denoted by arrows. On each device ($3\times3$ arrays), four grating units are fabricated for measuring S_1_ and S_2_, as well as two integrated silicon metasurface units on gratings with perpendicular orientations for measurement of S_3_ on top right and bottom right (the other two units on top and bottom left are designed for device demonstration around another central wavelength). S_0_ can be measured on the bare substrate. The central unit corresponds to gold mirror for focus alignment of objective during FTIR measurement. The scalebars for gratings and silicon-nanopillar integrated units are 1 $\mu m$ and 500 nm, respectively.

## Supplementary references

1 Gansel, J. K. *et al.* Gold helix photonic metamaterial as broadband circular polarizer. *Science* **325**, 1513-1515, (2009).

2 Frank, B. *et al.* Large-Area 3D Chiral Plasmonic Structures. *ACS Nano* **7**, 6321-6329, (2013).

3 Zhang, M. *et al.* Nanoimprinted Chiral Plasmonic Substrates with Three-Dimensional Nanostructures. *Nano Letters* **18**, 7389-7394, (2018).

4 Chen, W., Abeysinghe, D. C., Nelson, R. L. & Zhan, Q. Experimental Confirmation of Miniature Spiral Plasmonic Lens as a Circular Polarization Analyzer. *Nano Letters* **10**, 2075-2079, (2010).

5 Decker, M., Klein, M. W., Wegener, M. & Linden, S. Circular dichroism of planar chiral magnetic metamaterials. *Opt. Lett.* **32**, 856-858, (2007).

6 Li, W. *et al.* Circularly polarized light detection with hot electrons in chiral plasmonic metamaterials. *Nature Communications* **6**, 8379, (2015).

7 Dong, J., Zhou, J., Koschny, T. & Soukoulis, C. Bi-layer cross chiral structure with strong optical activity and negative refractive index. *Optics Express* **17**, 14172-14179, (2009).

8 Zhao, Y., Belkin, M. A. & Alù, A. Twisted optical metamaterials for planarized ultrathin broadband circular polarizers. *Nature Communications* **3**, 870, (2012).

9 Zhao, Y. *et al.* Chirality detection of enantiomers using twisted optical metamaterials. *Nature Communications* **8**, 14180, (2017).

10 Zhu, A. Y. *et al.* Giant intrinsic chiro-optical activity in planar dielectric nanostructures. *Light: Science &Amp; Applications* **7**, 17158, (2018).

11 Hu, J. *et al.* All-dielectric metasurface circular dichroism waveplate. *Scientific Reports* **7**, srep41893, (2017).
